# Comparison of first-time microvascular decompression with percutaneous surgery for trigeminal neuralgia: long-term outcomes and prognostic factors

**DOI:** 10.1007/s00701-021-04793-4

**Published:** 2021-03-22

**Authors:** Imran Noorani, Amanda Lodge, Andrew Durnford, Girish Vajramani, Owen Sparrow

**Affiliations:** 1 Department of Neurosurgery, Wessex Neurological Centre, University Hospital Southampton, Tremona Road, Southampton, SO16 6YD UK; 2 Department of Neurosurgery, Addenbrooke’s Hospital, Cambridge University Hospitals NHS Foundation Trust, Cambridge, CB2 0QQ UK

**Keywords:** Microvascular decompression, Balloon compression, Glycerol, Thermocoagulation, Trigeminal neuralgia, Pain

## Abstract

**Objective:**

Common surgical treatments for trigeminal neuralgia (TN) include microvascular decompression (MVD) and percutaneous procedures (glycerol rhizolysis; thermocoagulation; and balloon compression). Although the efficacy of each procedure has been documented, direct comparisons of their relative efficacies for TN are lacking. We aimed to directly compare long-term outcomes after first-time MVD with percutaneous surgery in primary (idiopathic and classical) TN and identify predictors of outcome.

**Methods:**

We conducted a retrospective analysis of prospectively collected data on 185 patients undergoing MVD and 129 undergoing percutaneous surgery. Procedures were performed by one of two neurosurgeons in a single centre; an independent observer collected long-term follow-up data by interviews, using the same outcome measures for all procedures.

**Results:**

MVD patients were younger than those undergoing percutaneous surgery (*P* <.001). MVD provided superior initial pain relief (*P* <.001): 87.0% had Barrow Neurological Institute class I or II pain scores after MVD compared with 67.2% after percutaneous surgery. The complication rate for percutaneous procedures was 35.7% and for MVDs was 24.9% (*P* =.04), including minor and transient complications. Kaplan-Meier analysis demonstrated that MVD provided longer pain relief than percutaneous procedures (*P* <.001); 25% of patients had recurrence at 96 months following MVD compared with 12 months after percutaneous surgery. Subgroup analysis showed that balloon compression provided more durable relief amongst percutaneous procedures. Multivariate analysis revealed that post-operative numbness and age were prognostic factors for percutaneous procedures (*P* =.03 and .01, respectively).

**Conclusions:**

MVD provides better initial pain relief and longer durability of relief than percutaneous surgery, although carrying a small risk of major complications. Amongst percutaneous procedures, balloon compression gave the most durable relief from pain. Older age and post-operative numbness were predictors of good outcome from percutaneous surgery. These results can help clinicians to counsel patients with primary TN on neurosurgical treatment selection for pain relief.

**Supplementary Information:**

The online version contains supplementary material available at 10.1007/s00701-021-04793-4.

## Introduction

Trigeminal neuralgia (TN) is a syndrome of paroxysmal, severe facial pain in the trigeminal nerve sensory distribution. The more definitive treatment for TN is microvascular decompression (MVD) to relieve the causative physical compression of the trigeminal nerve by a neighbouring blood vessel [12, 11]. Other surgical treatments are ablative, aiming to injure the nerve to improve pain, and include three percutaneous procedures (glycerol rhizolysis, GR; thermocoagulation, TC; balloon compression, BC) and stereotactic radiosurgery (SRS).

The long-term relief rates for MVD have been reported to range between 70 and 80% at 5–10 years in several large series [4, 42, 52]. The relief rates for percutaneous surgery for TN are more variable and influenced by surgical technique and patient factors [6, 20, 28, 44, 46, 34, 35]. Comparing between studies is often challenging owing to the heterogeneity in outcome measures employed. According to a recent classification, there are three diagnostic categories of TN: ‘idiopathic TN’ is primary TN without any identifiable causes after appropriate investigations [9], ‘classical TN’ is primary TN with neurovascular compression of the trigeminal nerve root demonstrated on imaging, and ‘secondary TN’ is TN due to an underlying confirmed neurological disease. Although many studies report outcomes for individual types of procedure, few studies have directly compared MVD with percutaneous procedures in terms of long-term outcomes while controlling for potentially confounding patient factors [48, 49] such as atypical TN, recurrent pain or repeat surgery, and multiple sclerosis (MS). Therefore, a direct comparison of MVD versus percutaneous procedures with long-term outcomes, in the context of first-time surgery for patients with primary (classical or idiopathic) TN, would be important in guiding clinicians on neurosurgical decision-making and counselling TN patients on surgical options.

Here, we performed a retrospective analysis of prospectively collected data from a large patient cohort, comparing long-term outcomes of MVD with percutaneous procedures (GR, TC, and BC) in a relatively homogenous population of primary (idiopathic and classical), typical TN patients undergoing first-time surgery. All procedures were performed by only two experienced neurosurgeons in a single centre, and an independent observer collected long-term follow-up data with the same outcome measures to minimise biases.

## Methods

### Patient characteristics

**Fig. 1:**
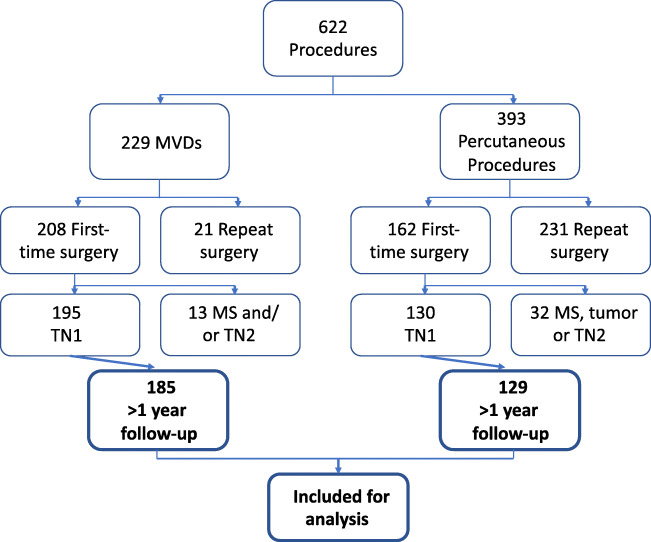
Flow chart showing the number of patients included in this study. ‘TN1’, classical or idiopathic, type 1 trigeminal neuralgia; ‘TN2’, atypical, type 2 trigeminal neuralgia

This study had patient consent and institutional ethical board approval. Clinical data for all consecutive patients who underwent surgery for TN in University Hospital Southampton were prospectively collected. Between 1996 and 2012, 622 surgical procedures were performed for TN; 185 patients who had MVD and 129 who had percutaneous surgery (54 GR, 49 TC, and 26 BC) were included in this study (Fig. 1 and Table 1). Inclusion criteria were idiopathic or classical TN without MS or tumours (appropriate investigations including MRI were performed in all patients to exclude these) [9], type 1 TN, first-time surgery for TN, and more than 12-month follow-up. Ten patients who had an MVD were lost to follow-up; 1 patient with a percutaneous procedure (TC) was lost to follow-up (*P* = 0.055, 2-sided Fisher’s exact test). Cases with MS or repeat surgery have been reported separately [34, 35]. All patients in this work had typical (Burchiel type I [8]) TN pain, meeting the criteria of pain in the distribution of the trigeminal nerve (Vn); an intermittent, paroxysmal course; shock-like lancinating character; light-touch triggers; and initial response to TN medications. There were no exclusions from surgical treatment on purely age or comorbidity grounds. A pooled cohort analysis of all percutaneous TN procedures in our centre has been previously published [34].

**Table 1:** Patient demographics categorised according to procedure type. *MVD* microvascular decompression, *GR* glycerol rhizolysis, *TC* thermocoagulation, *BC* balloon compression

			Percutaneous subgroups		
Variable	MVD	Percutaneous surgery	GR	TC	BC
Procedures, *n*	185	129	54	49	26
Male procedures, *n*	88	50	24	19	7
Female procedures, *n*	97	79	30	30	19
Age (mean), year	58.5	72.7	70.9	74	73
Age range, year	24.3–83.0	43.5–93.7	46.8–88.7	44.0–92.0	51.0–94.0
Side (L:R)	0.43:0.57	0.43:0.57	0.50:0.50	0.41:0.59	0.35:0.65
V1 only, %	7.3	2.5	5.9	0	0
V2 only, %	21.3	16.4	5.9	25	21.7
V3 only, %	17.7	18	9.8	25	21.7
V1 and V2, %	18.9	19.7	31.4	4.2	26.1
V2 and V3, %	20.1	31.1	27.5	41.7	17.4
V1–3, %	14.6	12.3	19.6	4.2	13

### Data collection and patient follow-up

MVD, GR, and TC have been employed in our centre for over 20 years, whereas BC was introduced in 2010 after the appointment of a second neurosurgical pain consultant (GV). Patient data were collected from operation notes and clinic letters. Routine clinic follow-up of these patients was conducted at 3 months and 18 months after their operation. Additional telephonic follow-up was conducted and/or questionnaires were completed to obtain long-term data. An independent, unbiased research doctor (I.N.) or specialist TN nurse (A.L.) performed these long-term interviews, minimising potential reporting biases from patients being reluctant to disappoint their treating clinician by directly reporting poorer outcomes to them. Follow-up time extended up to 20 years (minimum 1 year; mean 51.8 months).

Initial pain relief (3 months post-procedure) was defined according to the Barrow Neurological Institute (BNI) pain intensity score [41]: ‘class I, no pain and no analgesic treatment needed; class II, occasional TN pain not requiring medication; class III, some TN pain that is adequately controlled on medication; class IV, some TN pain, not adequately controlled on medication; and class V, severe TN pain, with minimal/no relief from the procedure’. Time to recurrence after a procedure was defined as time between the operation and restarting of severe TN pain not adequately controlled by medication and/or needing re-operation. New facial numbness post-procedure was classified as none/mild, moderate, and dense, with an additional category of ‘bothersome’ numbness that was counted as a complication due to its intrusive nature. Sensory disturbance was assessed by physical examination at outpatient visits and patients’ own reports at telephone interview on how they themselves assessed the numbness.

The reporting period began before the availability of cisternography using fine-cut MRI with gradient echo sequences (3D constructive interference in steady state, CISS), which became available in our centre from the year 2000; prior to this, lower resolution MRI images with MR angiography alone were used from a 0.5-T scanner. Pre-operatively all MR films were assessed for neurovascular compression (NVC) by the consultant neurosurgeon and a consultant neuroradiologist.

### Selection of procedure

All operations were performed by 1 of 2 consultant neurosurgeons (G.V. and O.S.) directly or under their personal supervision. Both surgeons were fully trained and well-experienced in all procedures prior to operating on cases for this study. The decision-making strategy we used for selecting operations for individual patients is in line with published algorithms [1, 45]. MVD was offered to all patients with TN who were medically fit and relatively young, and where the MRI scan suggested NVC. All these patients were additionally counselled for a partial sensory rhizotomy (PSR), as a ‘fall-back’ position if no convincing NVC was found at operation. When NVC was observed intra-operatively, an MVD was completed; otherwise the operation would be converted to a PSR; this occurred in only 3 cases. Where the MRI did not suggest NVC, patients were offered the option of a percutaneous lesion or (if medically fit and young) exploration with MVD or probable PSR. For patients who were less medically fit, older, and/or unwilling to undergo a larger, longer operation, a percutaneous procedure was offered. Given this, the majority of patients undergoing MVD had classical TN (with NVC demonstrated on imaging), whereas those undergoing percutaneous surgery were a more mixed population of idiopathic TN and classical TN. Patients with significant V1 pain were not offered TC due to a risk of corneal anaesthesia. All patients were counselled on all procedures, with the final choice of procedure largely dependent on individual patient preference. All patients were interviewed face-to-face in an outpatient setting by the consultant surgeon to confirm diagnosis and discuss treatment options. Information leaflets were available, as was telephonic contact with a specialist nurse who was generally present during the initial consultation.

The vast majority of patients offered MVD accepted it. A few were unsuitable due to comorbidity, and most without NVC (mainly early in the series, before the quality of MR had been improved) offered an exploration similarly agreed, expecting a partial sensory rhizotomy if exploration was negative. Patients over 85 years old were excluded for MVD as none was medically suitable. All cases up to 75 years were offered open surgery when fit, as were those up to 80 in reasonable health, and only the exceptionally fit cases aged over 80 years were offered open surgery. Many of the elderly patients however preferred a shorter ‘day-case’ percutaneous procedure, declining open surgery. Records of exact numbers of patients who were offered surgery but declined were not available; however, the senior author is confident of small numbers for those with probable NVC and medically fit who declined MVD, and for those without NVC and offered exploration, who declined this option. Similarly, we do not have records for the exact number of patients with probable NVC who were not offered MVD due to age/medical comorbidities.

Although SRS was not available at our centre, patients were referred for SRS to another centre where mass lesions of Meckel’s cave/cavernous sinus could be simultaneously treated by this modality (two patients), or critical anticoagulation ruled out a percutaneous procedure requiring even only temporary reversal (one patient). In these cases, there was no need for immediate pain relief. However, due to limited facilities for SRS in the UK during this study period and geographical distance of these from our centre, SRS was not offered as a ‘surgical’ first-line in primary TN.

### Operative technique

Percutaneous procedures were performed under general anaesthesia or sedation with fluoroscopic guidance, with minimal variation in technique between cases. For GR, a 20-gauge needle was inserted into the medial foramen ovale following Härtel’s technique, and advanced deep toward the clivus on a lateral projection. After reversal of anaesthesia, contrast was injected with the patient in the sitting position and head flexed forwards; contrast was slowly replaced with glycerol (0.36 ml, the median volume of Meckel’s cave). The needle was then withdrawn, with patient position maintained for at least 2 h. For TC, a similar needle insertion technique was used, with the needle tip placed more laterally in the foramen ovale to reach the trigeminal ganglion. Electrode tip position was confirmed by awake testing before re-sedation and heating the electrode to between 65 and 70°C for 60 s. One thermal lesion was made, except where there was combined V2 and V3 neuralgia with evoked paraesthesiae in only one of these divisions, in which case a second lesion was performed for the other division following needle adjustment and re-testing.

Following the same needle insertion technique via the middle of the foramen ovale on its posterior rim, with a soft Fogarty balloon and minor test inflation with contrast to confirm correct balloon configuration, Vn compression entailed balloon inflation with 0.35–0.50 ml of contrast to obtain a ‘pear’ shape (known to improve pain outcomes [33]), with three alternating cycles of 60s of inflation and 60s of deflation.

For MVD, the operative technique was similar to that of published techniques [5, 18, 31]. Following a small retrosigmoid craniotomy, Vn was inspected under the microscope for vascular compression anywhere along its course. Arteries causing compression were dissected from the trigeminal root and then padded with shredded Teflon felt, or the vessels were elevated in a Teflon sling sutured to the tentorium. Patency of the superior cerebellar artery past the sling was confirmed using either micro-Doppler or, more recently, intra-operative indocyanine green angiography. Compressive veins were coagulated and divided. No adverse events followed vein coagulation. The veins divided were usually transverse, draining to the superior petrosal vein, or in one or two, the superior petrosal vein itself. Neurovascular compression of Vn was noted intra-operatively using a published grading system [27]: grade I, mere contact with Vn; grade II, displacement or distortion of the nerve root; and grade III, marked indentation of Vn.

### Statistical analysis

Statistical analysis was performed using IBM SPSS 25 (IBM Analytics, New York) software, with a significance level of *P* < .05 for all tests. The 2-sided chi-square test or Fisher’s exact test was used to compare proportions between groups of patients. Kaplan-Meier curves were compared by the log-rank test. Data were censored if there was no pain recurrence at the most recent follow-up. To identify predictors of pain relief, univariate analysis was performed by logistic regression; multivariate logistic regression was performed using those variables exhibiting a *P* < .2 from univariate analysis.

## Results

Patients undergoing MVD were significantly younger than those undergoing a percutaneous procedure (mean age 58.5 years vs 72.7 years, respectively; *P* < .001, unpaired two-tailed *t*-test; range 24.3–83.0 years for MVD and 43.5–93.7 years for percutaneous procedures) and had a shorter duration of pre-operative TN pain (mean 7.05 years compared with 9.14 years, respectively; *P* = .022, two-tailed *t*-test).

### Initial relief

**Fig. 2:**
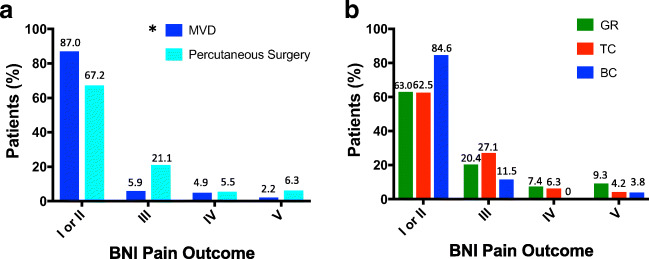
Initial pain relief outcomes, reported using the Barrow Neurological Institute (BNI) pain scale, at 3 months. **a** A plot of BNI pain scores after MVD and percutaneous surgery; ‘*’ denotes significantly better BNI scores after MVD compared with percutaneous procedures (*P* < .001). **b** A plot of outcome scores after percutaneous surgery classified according to procedure type. ‘GR’, glycerol rhizolysis; ‘TC’, thermocoagulation; ‘BC’, balloon compression

For MVD, 87.0% of operations yielded class I or II pain relief at 3 months, in comparison with 67.2% of patients who had a percutaneous procedure. Only 7.0% of patients who had undergone MVD and 11.7% of patients who had undergone a percutaneous procedure had little or no relief after their operation (BNI class IV and V). Comparing the distributions of initial pain relief demonstrated that MVDs provided significantly better initial relief compared with percutaneous procedures (2-sided chi-square test, *P* < .001; Fig. 2a).

Subgroup analysis showed that initial class I or II relief rates for GR, TC, and BC were 63.0%, 62.5%, and 84.6%, respectively. Little or no pain relief (class IV or V) was achieved after 16.7%, 10.4%, and 3.8% of GR, TC, and BC procedures, respectively. MVD provided significantly better initial relief than GR and TC (*P* < .001 for both MVD vs GR and MVD vs TC, chi-square test) but not compared with BC when comparing the distributions of pain relief (*P* = .45, chi-square test). There were no significant differences in distributions of initial pain relief scores between GR, TC, and BC (GR vs TC, *P* = .68; GR vs BC, *P* = .21; TC vs BC, *P* = .20, chi-square test; Fig. 2b).

### Complications

**Fig. 3:**
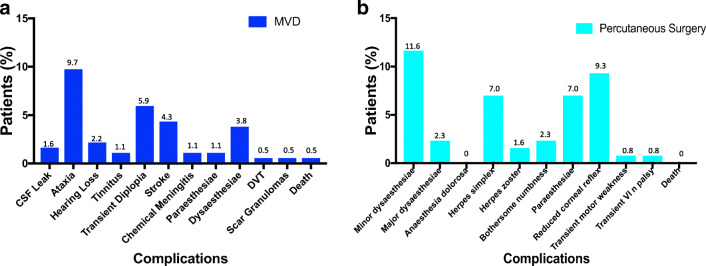
Plots of complication rates after MVD (**a**) and percutaneous surgery (**b**). For **b**, motor weakness and VIn palsy after percutaneous surgery were transient

Following MVD, there were 3 (1.6%) cases of CSF leak requiring temporary CSF diversion with a lumbar drain, 8 (4.3%) cases of minor stroke (typically some hemisensory loss and mild weakness that improved), 2 (1.1%) cases of chemical meningitis that recovered, and 1 (0.5%) case of deep venous thrombosis (DVT). Two (1.1%) patients reported facial paraesthesiae and 7 (3.8%) reported dysaesthesiae. There was one (0.5%) death after an MVD due to a post-operative posterior fossa haematoma in a patient with underlying but peri-operatively treated coagulopathy. The commonest complications were minor: ataxia (9.7%) and diplopia (5.9%) which improved (Fig. 3a).

After a percutaneous procedure, there were 15 (11.6%) cases of minor dysaesthesiae and 3 (2.3%) of major dysaesthesiae, 3 (2.3%) of bothersome numbness, 12 (9.3%) of reduced corneal reflex, and 9 (7.0%) of facial paraesthesiae (Fig. 3b). There were no deaths or cases of anaesthesia dolorosa. Complications stratified by type of percutaneous procedure are indicated in Supplementary Table 1. The overall complication rate (including minor and transient complications) for MVD was 24.9% and for percutaneous procedures was 35.7% (*P* = .04, Fisher’s exact test).

### Numbness

**Fig. 4:**
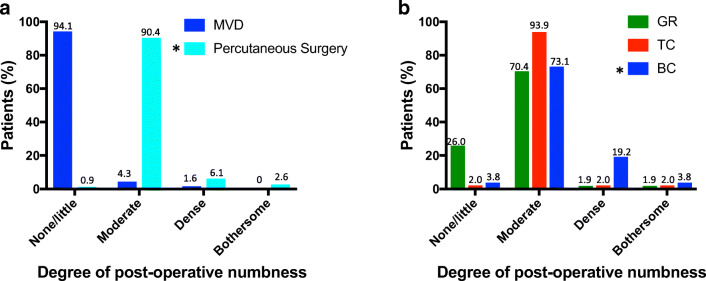
Post-operative numbness. **a** A plot showing the degree of post-operative numbness after MVD and percutaneous surgery. ‘*’ denotes a significantly higher degree of dense or bothersome numbness after percutaneous surgery than MVD (*P* = .006). **b** A plot of post-operative numbness after percutaneous surgery, categorised according to procedure type, showing a significantly higher degree of dense/bothersome numbness after BC than GR or TC (*P* = .05 and .02, respectively)

At 3-month follow-up following MVD, 4.3% of patients had mild to moderate facial numbness, and 1.6% had dense numbness; there were no cases of bothersome numbness. In contrast, 90.4% of percutaneous cases developed mild to moderate numbness, and there were significantly more cases of dense (6.1%) and bothersome (2.6%) numbness than with MVD (*P* = .006, Fisher’s exact test; Fig. 4a).

Subgroup analysis of percutaneous procedures showed that BC generated a greater proportion of cases with dense or bothersome post-operative numbness than GR and TC (23.1% in BC, 5.0% in GR, and 4.2% in TC; BC vs GR, *P* = .05; BC vs TC, *P* = .02, Fisher’s exact test; Fig. 4b). There was no significant difference in the proportion of cases with dense/bothersome numbness with GR compared with TC (*P* = 1.00, Fisher’s exact test).

### Durability of pain relief

**Fig. 5:**
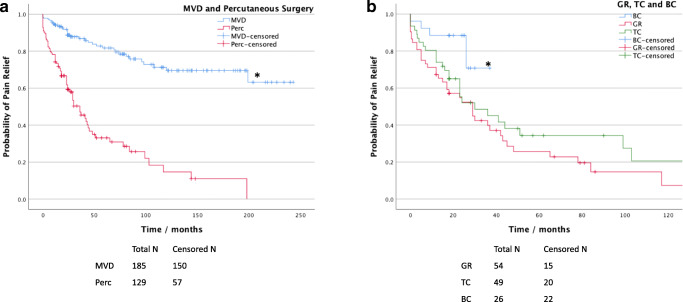
Long-term pain relief after first-time surgery for idiopathic TN. **a** Kaplan-Meier curves of time to pain recurrence for MVD and all percutaneous procedures. ‘*’ denotes significantly better durability of relief with MVD than percutaneous surgery (*P* < .001). ‘Perc’ denotes percutaneous procedures. **b** Kaplan-Meier curves of GR, TC, and BC, showing significantly higher durability of relief with BC than GR or TC (*P = .011 and .031, respectively)

Kaplan-Meier (KM) analysis for time to recurrence of TN demonstrated that MVD provided significantly longer pain relief compared with percutaneous procedures overall (*P* < .001, log-rank test; Fig. 5a). The median time to pain recurrence for percutaneous procedures was 36 months (95% confidence interval, CI, 24.9–47.1 months) and was not yet reached for MVDs; 25% of patients had pain recurrence at 96 months following an MVD compared with only 12 months after a percutaneous procedure.

Subgroup analysis confirmed that the durability of pain relief for MVD was significantly better compared with GR and TC (*P* < .001 for both MVD vs GR and MVD vs TC, log-rank test), and non-inferior to BC (*P* = .27, log-rank test). The median time to pain recurrence was 29 months (95% CI 16.1–41.9 months) for GR, 30 months (95% CI 8.6–51.4 months) for TC, and not yet reached for BC. Twenty-five percent of patients had pain recurrence at 5 months following a GR, 12 months after a TC, and 26 months after a BC. Although there was no difference in durability of relief after a GR compared with TC (*P* = .24, log-rank test), BC provided significantly longer pain relief compared with GR and TC (*P* = .011 for BC vs GR; *P* = .031 for BC vs TC, log-rank test; Fig. 5b).

### Predictors of pain relief

For MVD, age, sex, pre-operative duration of TN pain, and trigeminal division of pain were not significant predictors of favourable outcome after surgery (as defined by initial post-operative BNI score I or II *and* no recurrence on long-term follow-up) using univariate analysis. With pre-operative MRI findings of neurovascular contact or compression of Vn, there was a favourable outcome in more cases (*P* = .10, OR 2.48, 95% CI 0.83–7.42), and similarly for severe intra-operative NVC (grade II or III; *P* = .06, OR 2.12, 95% CI 0.97–4.65) on univariate analysis (Table 2); these differences were not statistically significant. The type of compressive blood vessel (artery or vein) was not associated with outcome. Multivariate logistic regression analysis revealed no significant predictors of outcome after MVD. The causative vessel compressing Vn was observed to be the superior cerebellar artery in 67.2%, anterior inferior cerebellar artery in 4.5%, multiple arteries in 8.1%, an artery and a vein in 11.6%, and a vein only in 8.6% of cases.

**Table 2:** Univariate and multivariate logistic regression analysis for predictors of outcome for MVD. Note that ‘MRI NV contact/distortion’, ‘mixed arterial and venous compression’, and ‘severe NVC’ are inter-related (multi-collinear) variables, and therefore only one of each was included per model in the multivariate analysis in conjunction with ‘right side’ [40]. Predictors giving a probability value 0.05 < *P* < .10 are in italics

Variable	Univariate analysis				Multivariate analysis			
		95% CI				95% CI		
	Odds ratio	Low	High	*P*	Odds ratio	Low	High	*P*
Age	0.995	0.966	1.02	.72				
Male sex	1.587	0.779	3.233	.20				
Right side	1.723	0.862	3.446	.12	1.603	0.794	3.233	.19
V1 pain	0.966	0.465	2.006	.93				
V2 pain	0.659	0.276	1.577	.35				
V3 pain	0.737	0.356	1.524	.41				
Pre-operative TN duration (years)	1.036	0.972	1.103	.27				
MRI NV contact/distortion	2.481	0.829	7.429	.10	2.481	0.821	7.500	.11
Venous compression only	0.561	0.18	1.742	.32				
Arterial compression only	1.22	0.552	2.694	.62				
Mixed arterial and venous compression	3.279	0.734	14.647	.12	3.253	0.724	14.607	.124
Post-operative numbness	0.77	0.195	3.044	.71				
Severe NVC	2.121	0.967	4.65	*.06*	2.003	0.907	4.421	.086

Univariate logistic regression confirmed that, for all percutaneous procedures combined, sex, side and distribution of TN pain, and pre-operative duration of TN symptoms were not predictors of outcome. Older age and the presence of post-operative dense or bothersome numbness were the only significant predictors of favourable outcome (*P* = .007, OR 1.06, 95% CI 1.02–1.10 for age; *P* = .02, OR 5.17, 95% CI 1.27–21.12 for post-operative numbness). On multivariate logistic regression analysis, both age and post-operative numbness remained significant predictors of outcome (*P* = .01 and .03, respectively; Table 3).

**Table 3:** Univariate and multivariate logistic regression analysis for predictors of outcome (as defined by initial post-operative BNI score I or II *and* no recurrence on long-term follow-up) for percutaneous surgery. Significant predictors are in bold (*P* < .05)

Variable	Univariate analysis				Multivariate analysis			
		95% CI				95% CI		
	Odds ratio	Low	High	*P*	Odds ratio	Low	High	*P*
Older age	1.056	1.015	1.098	**.007**	1.055	1.014	1.098	**.008**
Male sex	0.739	0.346	1.577	.43				
Right side	1.347	0.641	2.827	.43				
V1 pain	1.634	0.763	3.499	.21				
V2 pain	1.5	0.603	3.732	.38				
V3 pain	0.641	0.306	1.342	.24				
Pre-operative TN duration (years)	0.995	0.951	1.041	.83				
Dense/bothersome post-operative numbness	5.171	1.266	21.12	**.022**	5.106	1.187	21.972	**.029**

## Discussion

Published outcomes of MVD and ablative treatments for TN vary due to differences in surgical technique, outcome measures, patient selection, and study methods; most studies report results for single techniques. In this large retrospective analysis, we compare the long-term outcomes of MVD with three types of percutaneous procedure in primary (classical or idiopathic) TN, using the same outcome measures. Such results have rarely been reported together for direct comparisons [19, 26, 13, 23, 15]. Key advantages of this study over prior publications are the prospective data collection of a large dataset, long-term follow-up by an independent observer (reducing reporting bias), and performance of all procedures at a single centre by only two neurosurgeons (mitigating surgical heterogeneity). These advantages enhance the reliability of the findings reported here. Our results indicate higher long-term pain-free rates in patients treated with MVD compared with percutaneous procedures, with balloon compression giving better pain relief amongst percutaneous procedures.

The efficacy of MVD in our study, with 87% of patients achieving initial BNI class I or II pain scores and 25% of patients having pain recurrence by 96 months, is similar to published observations [39, 38, 4, 43]. Wang et al. reported a median time to recurrence of 94 months, with 25% of patients having a recurrence at 57 months [52]; Barker et al. found that 70% of patients had class I relief 10 years after an MVD [4], similar to Sarsam et al. (71%) [42]. In comparison, 67% of our patients had initial class I or II pain relief after a percutaneous procedure, with the median time to recurrence of 36 months. Prior studies report excellent initial pain relief in 59–91% of patients after GR [51, 36], 80–100% after TC [47, 51], and 82–91% after BC [25, 2, 30]. These results are consistent with our data in which GR and BC produced class I or II pain scores in 63% and 85%, respectively; the lower TC initial pain-free rate (63%) reported here compared with other studies may reflect the cautious technique (with a single thermal lesion) we employed, knowing that the lesion could be repeated in the event of recurrence.

Previous work has suggested a correlation between sensory loss and pain relief following destructive procedures for TN [11, 7], which would be explained by more destructive lesions giving more durable relief. In line with this, we found that post-operative numbness is a significant predictor of favourable outcome for percutaneous procedures but not for MVD and is also a significant predictor for lack of recurrence in long-term follow-up. Moreover, BC produced a higher level of post-operative numbness, and this coincided with greater durability of relief compared with GR and TC. There was also a higher complication rate with BC compared with the other techniques and previous reports, although most of these complications were minor sensory disturbances such as paraesthesiae and minor dysaesthesiae. In our experience, most patients are willing to accept a degree of numbness and/or sensory disturbance post-operatively if they achieve adequate pain relief from TN. The technique we employed, with three cycles of 60 s of balloon compression, is likely to have a stronger destructive effect than a single cycle of compression reported in some studies [24, 3], thus explaining these results. To our knowledge, age has not been reported as a predictor of outcome from percutaneous surgery in other series. Age had a weak effect according to our data. It remains to be confirmed in other large series whether advancing age provides a ‘true’ benefit for efficacy of these procedures, or whether this is due to older patients possibly being more stoical and less likely to request further surgery.

Previously reported prognostic factors for MVD include male sex, arterial compression, duration of TN pain, and typical TN [14, 16, 32, 50]. Our analysis demonstrated more patients with neurovascular contact/compression seen on MRI and severe NVC observed intra-operatively to had better outcomes after MVD, although this difference was not statistically significant. However, the reporting period of this study started before the availability of high-resolution MRI with gradient echo sequences, which has a higher sensitivity for detecting NVC pre-operatively. Some previous studies have proposed that the severity of NVC is important in determining the success of MVD [17, 37], although others have not [52]. Other than physical compression, inflammation and demyelination have been proposed as pathophysiological causes of TN [29, 10], explaining why decompressing Vn may not be enough to resolve TN in certain cases [21].

MVD and percutaneous procedures have advantages and limitations. While MVD provides superior long-term pain relief overall, it is a larger and longer operation than percutaneous procedures, requires longer hospital stay, and carries a risk (albeit low) of serious complications. Patients with ischaemic stroke after MVD in this series had relatively mild neurological deficits that improved. The minor strokes were assumed to be due to disruption or kinking of small brainstem perforators during the mobilisation of the caudal loop of the superior cerebellar artery from deep to the trigeminal nerve root. Most minor strokes were revealed by direct enquiry of patients during routine follow-up, by which time they had recovered fully. In some early MVD cases, polyvinyl alcohol (Ivalon) foam was used and resulted in two cases of chemical meningitis, and thus we decided to use Teflon felt subsequently. In the patient who unfortunately died after MVD due to a cerebellar haematoma, no vein was coagulated intra-operatively, and their pre- and intra-operative correction of modest coagulopathy associated with chronic myeloma was undertaken on haematological advice.

Percutaneous procedures are less invasive, simpler to perform, need shorter hospital stay (typically not overnight), and can also provide immediate relief. However, percutaneous procedures generally provide shorter durability of relief compared with MVD (although can be effectively repeated [34]) and have a higher risk of dysaesthesiae. Although the complication rate for percutaneous procedures was higher than MVD here, these complications were more minor. Similarly, a large proportion of MVD complications were minor and transient, including ataxia and diplopia. SRS is an alternative, minimally invasive treatment for TN, which has a destructive effect on Vn. It is used in cases where a longer latency between treatment and pain relief is tolerated and/or for those unable to undergo invasive surgery [22].

## Limitations

Limitations of this study include the retrospective nature of the analysis (although most surgical TN literature is retrospective) [53]. Patients were not randomly assigned to a procedure, and there was no blinding of procedure to clinicians or patients. Indeed, there is a major need for more randomised controlled trials in neurosurgical treatment of TN [53], though randomisation poses its own challenges. There was a mixture of patients with idiopathic and classical (primary) TN included here, and further studies focusing on each category separately would be beneficial. There were both longer follow-up and more cases undergoing MVD, GR, and TC compared with BC; with further follow-up of BC in the future, it will be useful to determine likely differences between durability of relief for MVD and BC in the longer term. Nevertheless, the primary aim of this study was to compare MVD with all percutaneous destructive procedures, as such studies are lacking. There were differences in baseline characteristics (age and duration of TN pain) between MVD and percutaneous surgery cohorts, which may have possibly contributed to differences in outcome, though older age in fact seemed to favour ablative procedures. SRS was performed in only one idiopathic TN case (and two cases with underlying tumours), so this selection bias was minimal. Our results apply only to primary (idiopathic and classical), type I TN, and not to atypical TN, MS, or recurrent pain after previous surgery.

## Conclusions

Surgical decision-making needs tailoring to the individual patient and depends on many factors. Our large single-centre analysis of first-time surgery for idiopathic and classical (primary) TN shows that MVD provides superior initial quality of relief and also durability of pain relief than percutaneous surgery, although carrying a low risk of major complications. Percutaneous procedures, particularly BC with the reported parameters, provide good pain relief with largely minor complications in patients less suitable for or unwilling to have a major operation. Older age and post-operative numbness are prognostic factors for better outcome after percutaneous procedures. We hope our data will aid surgeons in counselling TN patients for selecting between these procedures, and we propose these guidelines for first-time surgery in primary TN (based on surgical parameters employed here):

1. For younger and medically fit patients, MVD should be offered.
2. For older, less medically fit patients or those unwilling to undergo an open operation, balloon compression is suggested as first-line.
3. If balloon compression is not possible, glycerol rhizolysis or thermocoagulation can be offered, although thermocoagulation should be avoided in those with pure V1 pain.

## Supplementary information

ESM 1 (DOCX 14.7 kb)
