# Supplementary material for: Comparison of first-time microvascular decompression with percutaneous surgery for trigeminal neuralgia: long-term outcomes and prognostic factors
Source: Acta Neurochir (Wien). 2021 Mar 22;163(6):1623–34. doi: 10.1007/s00701-021-04793-4 (PMC8116280; doi:10.1007/s00701-021-04793-4)
Supplement: Supplementary file 1 — (DOCX 14.7 kb) [file 701_2021_4793_MOESM1_ESM.docx]

| **Complication Type** | **Percutaneous procedure, complications (%)** | | |
| --- | --- | --- | --- |
|  | **Glycerol Rhizolysis** | **Thermocoagulation** | **Balloon Compression** |
| Minor dysaesthesiae | 5.6 | 16.3 | 15.4 |
| Major dysaesthesiae | 1.85 | 4.1 | 0 |
| Anaesthesia dolorosa | 0 | 0 | 0 |
| Herpes simplex | 7.4 | 4.1 | 11.5 |
| Herpes zoster | 3.7 | 0 | 0 |
| Bothersome numbness | 1.9 | 2.0 | 3.8 |
| Paraesthesiae | 3.7 | 6.1 | 15.4 |
| Reduced corneal reflex | 9.3 | 4.1 | 19.2 |
| Motor (V) weakness | 0 | 0 | 3.8 |
| VI nerve palsy | 0 | 2.0 | 0 |
| Death | 0 | 0 | 0 |
| **Overall complication rate** | 31.5 | 32.7 | 50.0 |

**Supplementary Table 1. Complications following first-time percutaneous surgery for idiopathic TN, stratified by procedure type.** Motor weakness and Vin palsy after percutaneous surgery were transient. There were no cases of anaesthesia dolorosa.
